# Dental Technicians’ Perception of the Quality of Dentists’ Communication on the Fabrication of Removable Partial Dentures: A Cross-Sectional Study in Saudi Arabia

**DOI:** 10.7759/cureus.48245

**Published:** 2023-11-03

**Authors:** Hina Kausher, Mahesh Suganna, Abbasi Begum Meer Rownaq Ali, Aruna D. S, Sara Tarek Ahmed, Amit Punj, Abdurabu A Gomawi

**Affiliations:** 1 Department of Prosthodontics, Riyadh Elm University, Riyadh, SAU; 2 Department of Dentistry, Dental Public Health and Tobacco Cessation, Bengaluru, IND; 3 Department of Dentistry, Prosthodontic Residency Program, Montefiore Medical Center, The University Hospital for Albert Einstein College of Medicine, New York City, USA; 4 Department of Dentistry, Dental Laboratory, Dental University Hospital, King Saud University, Riyadh, SAU

**Keywords:** denture fabrication, saudi arabia, removable partial dentures, quality of dentists communication, dental technicians

## Abstract

Background: Effective and clear communication between the dentist and dental technician plays a vital role in rendering quality prostheses for patients. When fabricating a removable dental prosthesis, it is uncertain if the information received by the dental laboratory technician is clear and sufficient. This investigation aimed to assess dental technicians' perceptions of the quality of dentists' communication on the fabrication of removable partial dentures (RPDs) in Saudi Arabia.

Methodology: After obtaining the institutional review board (IRB) approval from Riyadh Elm University, Riyadh, Saudi Arabia, a cross-sectional survey on a convenient sample of 115 dental technicians registered with the Saudi Commission for Health Specialties was conducted in January 2022. The voluntary participation of 94 technicians fabricating RPDs was included. A 19-item online questionnaire was developed, including quality of written instruction, selection of impression trays, and impression materials for RPD fabrication, shared through Google Docs. Descriptive statistics were tabulated, and responses were displayed as a percentage of the total.

Results: Of the 94 study subjects, 35% had less than five years of experience, 44% stated that they routinely receive work authorizations with clear instructions, 13% always used digital technology to fabricate prostheses, and 58% reported difficulty with communicated work authorization by dentists having less than five years of experience. Thirty-three respondents (35.1%) reported that 75% or more of the fabricated partial dentures were cast framework partials. Thirty-three respondents (35.1%) indicated that the master casts received for partial framework construction were usually accurate. Tooth alterations, however, were reported as usually adequate by only 28 respondents (29.8%). For creating the artificial gingiva portion of cast partials, 56 respondents (59.6%) preferred heat-cured acrylic resin. Furthermore, 40 respondents (42.6%) said that 75% or more of the requested partials were entirely made out of acrylic resin. Regarding case design discussions, 26 respondents (27.7%) always engaged with dentists, while 39 (41.5%) did so occasionally.

Conclusion: The obtained assessments pointed to the fact that dental technicians expressed a perception of inadequacy regarding the work authorizations provided by dentists for the fabrication of RPDs, where they seemingly felt that the instructions conveyed by the dentists were not sufficiently comprehensive or clear.

## Introduction

Removable partial dentures (RPDs) remain an important treatment option for tooth loss [1]. Current research, however, lacks a focus on the relationship between dentists and dental laboratory technicians, especially about removable prostheses and the advancement of digital dentistry. Earlier studies have reported a decline in the hands-on laboratory work of dental school graduates [2]. Many dental schools do not require their students to complete all stages of denture fabrication. A significant number of these schools do not have in-house laboratories, leading to the need for collaboration with external dental laboratories [3].

Technology advancements have led to the rise of computer-assisted design and manufacturing methodologies in the creation of RPDs [3]. These include complete dentures and partial denture frameworks and use either printing or milling technology. However, traditional methods are still the mainstay for RPD design [4].

Over the past few decades, the dental laboratory industry has seen significant changes [5]. There has been a decrease in the number of dental laboratories due to consolidation into larger entities, the introduction of automated processes, and international competition [6]. There is also a reported decline in the number of skilled dental technicians, attributed to fewer accredited dental technology programs and retirements from the workforce [6].

Effective communication between the dentist and the dental technician is essential for delivering a high-quality prosthesis to the patient [7]. Lack of communication has been identified as a significant factor affecting the provision of optimal dental services [8-9]. This highlights the importance of understanding the roles and responsibilities of both parties.

Therefore, there is a need to reassess the standard of communication between dentists and dental laboratory technicians. This includes focusing on work authorizations and the choice of dental materials for the production of clinically acceptable RPDs, where work authorization in the context of our study refers to the approval or directive given to the laboratory to proceed with the fabrication of RPDs. It includes details about the specific work to be done, often provided by a dentist, after the needs of the patient have been assessed. Research in this area remains limited, particularly in certain regions [10]. Therefore, the primary objective of this investigation was to evaluate dental technicians' perceptions of the effectiveness of dentists' communication during the manufacture of RPDs in Saudi Arabia.

## Materials and methods

Study design

The described research was carried out within the geographical confines of Saudi Arabia, targeting professional dental technicians as the units of observation. A convenience sampling technique, a subtype of nonprobability sampling, was utilized. The study assured participants of voluntary involvement and guaranteed the confidentiality of the provided information. A cross-sectional, closed-ended survey was disseminated among the selected sample of experienced dental technicians in Saudi Arabia after the acquisition of approval from the Institutional Review Board of Riyadh Elm University, Riyadh, Saudi Arabia (approval number: FRP/2021/385/605/581), in December 2021.

Data collection

The survey instrument for the research was devised through a meticulous review of existing literature [9-11] and consultation with domain experts. It was specifically designed to elicit information pertaining to the clarity of written directives and the selection of impression trays and materials used in prosthesis fabrication. Written instructions were classified as: "Clear": the design instructions are clear and unambiguous; "A guide": most of the design instructions have been communicated; however, minor decision-making on the design has been left to the technician; "Poor": some of the design instructions have been communicated; however, the major decision-making on the design has been left to the technician; "None": no design instructions have been communicated.

The sample size was comprised of 112 study subjects, out of whom 94 responses were considered. The validity of the online questionnaire used in the study was tested by conducting a preliminary assessment on a randomly selected subject from the study population, representing 10% of the total. This process was carried out electronically, with participants accessing the questionnaire via a link generated by Google Docs (Google LLC, Mountainview, CA).

Inclusion and exclusion criteria

This investigation targeted a specific demographic of dental technicians registered as health practitioners with the Saudi Commission for Health Specialties. These practitioners operate across various domains, including academic institutions like dental colleges, dental clinics, and dental laboratories, both within the government and private sectors in Saudi Arabia, forming the sampling frame for this study. The inclusion criteria for the study were dental technicians actively engaged in the fabrication of RPDs and those who expressed a willingness to participate voluntarily while maintaining their confidentiality. The survey was disseminated electronically via a Google Docs link. The exclusion criteria included dental technicians not directly involved in the fabrication of RPDs and those who, upon outreach through email, opted not to participate in the study.

Statistical analysis

The data gathered were processed and examined using IBM SPSS Statistics Software for Windows, version 25.0 (IBM Corp., Armonk, NY). Descriptive statistics were initially used to provide a general overview of the data collected. This was followed by the application of inferential statistics.

## Results

Table 1 presents the findings of the cross-sectional analysis of the dental technicians' perceptions of the quality of communication with dentists.

**Table 1:** Baseline characteristics of the assessed population

Variable analysed	Responses	n	%
How many years has the lab been fabricating RPDs?	5 years or less	33	35.1%
	6 to 15 years	26	27.7%
	16 to 30 years	23	24.4%
	>30 years	12	12.8%
Are the technicians involved in the RPD fabrication laboratory-certified?	Yes	89	94.7%
	No	5	5.3%
What is the average work authorization received by the laboratory for the fabrication of RPDs?	Clear	41	43.6%
	A guide	36	38.3%
	Poor	15	16.0%
	None	2	2.1%
How often are work authorizations signed by the dentist?	Always (100% of the time)	38	40.4%
	Frequently (greater than 75% of the time)	35	37.2%
	Sometimes (25%-75% of the time)	16	17.0%
	Rarely (less than 25% of the time)	5	5.3%
How often does the laboratory use digital technology to design or fabricate RPDs?	Always (100% of the time)	12	12.8%
	Frequently (greater than 75% of the time)	22	23.4%
	Sometimes (25%-75% of the time)	14	14.9%
	Rarely (less than 25% of the time)	46	48.9%
Which age group of dental practitioners faces the most difficulty regarding quality of work and communication when fabricating an RPD?	New	55	58.5%
	Early	21	22.3%
	Mid	12	12.8%
	Senior	6	6.4%
Where are most of the RPDs fabricated on?	Semi-adjustable articulator with a facebow transfer	34	36.2%
	Semi-adjustable articulator without a facebow transfer	28	29.8%
	Simple hinge articulator without a facebow transfer	29	30.9%
	Others	3	3.2%

The majority of the laboratories surveyed (35.1%) had been fabricating RPDs for five years or less, followed by 27.7% of labs with six to 15 years of experience, 24.4% with 16 to 30 years, and 12.8% with over 30 years. The overwhelming majority (94.7%) of technicians involved in RPD fabrication were certified dental technicians, indicating a well-qualified workforce. The clarity of work authorization received for the fabrication of RPDs varied, with 43.6% of labs receiving clear instructions, 38.3% receiving a guide, 16% receiving poor instructions, and a minimal 2.1% receiving no instructions. However, the frequency of work authorizations signed by the dentist was found to be high, with 40.4% always getting them signed and 37.2% frequently getting them signed. Only a small fraction (5.3%) reported rarely getting it signed. The use of digital technology in RPD design or fabrication seemed less prevalent. Only 12.8% of labs reported always using digital technology, while 48.9% reported rarely using it. The frequency of its use was sometimes (14.9%) or frequently (23.4%) in the remaining labs. It was also observed that newer dental practitioners have the most difficulty regarding the quality of work and communication when fabricating an RPD, with 58.5% of respondents indicating this group. Early-career dentists followed with 22.3%, then mid-career dentists with 12.8%, and lastly senior dentists with 6.4%, indicating the least difficulty. The semi-adjustable articulator (Hanau™ Wide-Vue™ articulator, Whip Mix, Louisville, KY) with a facebow transfer (Hanau™ SpringBow, Whip Mix, Louisville, KY) was used most frequently (36.2%), followed by the simple hinge articulator without a facebow transfer (30.9%), and the semi-adjustable articulator without facebow transfer (29.8%). Only a small portion of labs (3.2%) reported using other methods.

**Figure 1:**
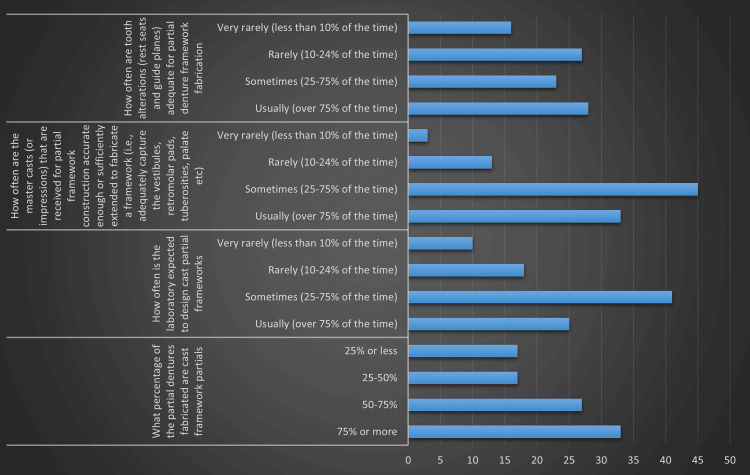
Graphical representation of characteristics regarding cast partial dentures

Table 2 and Figure 1 elaborate on the specifics of partial denture fabrication and the associated challenges.

**Table 2:** Characteristics of the cast partial dentures in the assessed population

Variable analysed	Responses	n	%
What percentage of the partial dentures fabricated are cast framework partials?	75% or more	33	35.10%
	50%-75%	27	28.70%
	25%-50%	17	18.10%
	25% or less	17	18.10%
How often is the laboratory expected to design cast partial frameworks?	Usually (over 75% of the time)	25	26.60%
	Sometimes (25%-75% of the time)	41	43.60%
	Rarely (10%-24% of the time)	18	19.10%
	Very rarely (less than 10% of the time)	10	10.60%
How often are the master casts (or impressions) that are received for partial framework construction accurate enough or sufficiently extended to fabricate a framework (i.e., adequately capture the vestibules, retromolar pads, tuberosities, palate, etc.)?	Usually (over 75% of the time)	33	35.10%
	Sometimes (25%-75% of the time)	45	47.90%
	Rarely (10%-24% of the time)	13	13.80%
	Very rarely (less than 10% of the time)	3	3.20%
How often are tooth alterations (rest seats and guide planes) adequate for partial denture framework fabrication?	Usually (over 75% of the time)	28	29.80%
	Sometimes (25%-75% of the time)	23	24.50%
	Rarely (10%-24% of the time)	27	28.70%
	Very rarely (less than 10% of the time)	16	17.00%
How often do dentists send good-quality opposing casts or impressions that can be articulated with or without an interocclusal record at the time of partial framework fabrication?	Usually (over 75% of the time)	40	42.60%
	Sometimes (25%-75% of the time)	41	43.60%
	Rarely (10%-24% of the time)	9	9.60%
	Very rarely (less than 10% of the time)	4	4.30%
What percentage of the frameworks that fit the master cast are returned by the dentist because they reported that the framework did not fit well in the mouth?	Over 20%	25	26.60%
	11%-20%	29	30.90%
	5%-10%	20	21.30%
	Less than 5%	20	21.30%
For mandibular distal extension partials, how often is it requested to perform the altered cast technique?	Usually (over 75% of the time)	19	20.20%
	Sometimes (25%-75% of the time)	30	31.90%
	Rarely (10%-24% of the time)	21	22.30%
	Very rarely (less than 10% of the time)	16	17.00%
	Never	8	8.50%
What type of resin and processing techniques are used for creating the artificial gingiva portion of cast partials (such as the flange area)?	Auto polymerizing acrylic resin (cold cured)	30	31.90%
	Heat-cured acrylic resin	56	59.60%
	Heat-cured injection molded technique.	8	8.50%

The distribution of cast framework partials was varied, with 35.1% of laboratories fabricating such partials in 75% or more cases, followed by 28.7% fabricating in 50%-75% of cases, and 18.1% each for the ranges of 25%-50% and 25% or less. This indicated a considerable practice of fabricating cast framework partial dentures across the laboratories. The frequency at which laboratories were asked to design cast partial frameworks was sometimes (43.6%) or usually (26.6%), with fewer laboratories being asked to do so rarely (19.1%) or very rarely (10.6%). This suggests that the design responsibility often fell on the laboratories. The accuracy of the master casts or impressions received was often a concern. While 35.1% of labs reported usually (over 75% of the time) receiving accurate casts, the majority (47.9%) reported only sometimes receiving accurate casts, with 13.8% rarely and 3.2% very rarely receiving accurate casts. The adequacy of tooth alterations for partial denture framework fabrication was found to be less than optimal. Only 29.8% of labs reported usually seeing adequate alterations, while 24.5% reported this sometimes, and a significant number of labs reported rarely (28.7%) or very rarely (17%) seeing adequate alterations. The quality of opposing casts or impressions sent by dentists was generally good, with 42.6% of labs reporting usually receiving good-quality casts and 43.6% reporting this sometimes. Only a small percentage reported rarely (9.6%) or very rarely (4.3%) receiving good quality casts. A notable 26.6% of labs reported over 20% of frameworks fitting the master cast being returned by the dentist due to poor fit in the mouth. This was followed by 30.9% reporting 11%-20% returns, and 21.3% each reporting 5%-10% and less than 5% returns. This may indicate discrepancies between the master cast and the actual oral cavity. Requests to perform the altered cast technique for mandibular distal extension partials varied, with 20.2% of labs usually receiving such requests, 31.9% sometimes, 22.3% rarely, and 17% very rarely. Interestingly, 8.5% never received such requests. Moreover, the type of resin used for creating the artificial gingiva portion of cast partials was predominantly heat-cured acrylic resin (59.6%), followed by auto-polymerizing acrylic resin (cold-cured) (31.9%), and heat-cured injection-molded technique (8.5%).

**Figure 2:**
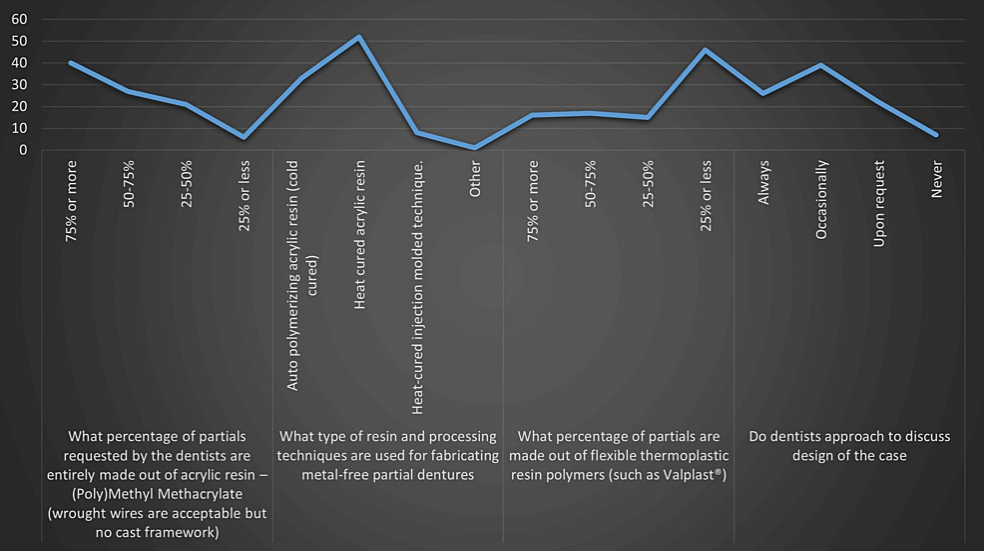
Graphical representation of characteristics regarding acrylic partial dentures and thermoplastic resin partial dentures

Table 3 and Figure 2 further delve into denture fabrication practices and communication between dentists and dental technicians.

**Table 3:** Characteristics regarding acrylic partial dentures and thermoplastic resin partial dentures in the assessed population

Variable analysed	Responses	n	%
What percentage of partials requested by the dentists are entirely made out of acrylic resin? (Poly)methyl methacrylate (wrought wires are acceptable but no cast framework)	75% or more	40	42.6%
	50%-75%	27	28.7%
	25%-50%	21	22.3%
	25% or less	6	6.4%
What type of resin and processing techniques are used for fabricating metal-free partial dentures?	Auto polymerizing acrylic resin (cold cured)	33	35.1%
	Heat-cured acrylic resin	52	55.3%
	Heat-cured injection molded technique.	8	8.5%
	Other	1	1.1%
What percentage of partials are made out of flexible thermoplastic resin polymers (such as Valplast®)?	75% or more	16	17.0%
	50%-75%	17	18.1%
	25%-50%	15	16.0%
	25% or less	46	48.9%
Do dentists approach to discuss the design of the case?	Always	26	27.7%
	Occasionally	39	41.5%
	Upon request	22	23.4%
	Never	7	7.4%

The distribution of partial dentures made entirely out of acrylic resin (poly)methyl methacrylate, allowing wrought wires but not cast framework, varied. The majority (42.6%) reported that 75% or more of the partials requested by the dentists were of this type, followed by 28.7% for the range of 50%-75%, 22.3% for 25%-50%, and a small percentage of 6.4% for 25% or less. This underlined a significant reliance on acrylic resin-based dentures. For fabricating metal-free partial dentures, the majority of laboratories employed heat-cured acrylic resin (55.3%), followed by auto-polymerizing acrylic resin (cold-cured) (35.1%), and heat-cured injection-molded technique (8.5%). Only 1.1% used other unspecified techniques. These results reflected a preference for heat-cured acrylic resin in metal-free partial denture fabrication. The use of flexible thermoplastic resin polymers, such as Valplast®, was less common. The majority (48.9%) reported 25% or less of partials being made from this material. This was followed by 18.1% for the range of 50%-75%, 17% for 75% or more, and 16% for 25%-50%. These findings indicated that while flexible thermoplastic resin polymers were used, they were not the predominant material choice. The communication between dentists and dental technicians, particularly about case design, varied. While 27.7% of technicians reported that dentists always approached them to discuss the design, a larger percentage (41.5%) indicated that this happened only occasionally. Furthermore, 23.4% reported being approached upon request, and 7.4% reported never being approached for case design discussions. These results highlighted the need for improved communication in denture fabrication, particularly in terms of case design.

## Discussion

This study reflects differences in dental technicians' perceptions of the quality of dentists' communication and standards of writing work authorizations for the fabrication of RPDs in Saudi Arabia. The assessments revealed a considerable discrepancy in the quality of impressions, the adequacy of tooth alterations, and the fitting of the final product. The study emphasized the significance of dental technicians in the process of case design and the necessity for enhanced dialogue between dentists and dental technicians. A substantial segment of the technicians had five years or less of hands-on experience, with a mere 13% possessing over 30 years of experience. This indicates a relatively smaller cohort of dental technicians who are adept with traditional laboratory procedures. This aligns with previous studies [12-13] that have noted a declining trend in the replacement rate of experienced dental technicians who retire, compounded by a decrease in the number of accredited dental laboratory training programs.

When asked about the average quality of work authorizations received for removable prosthetics, 43.6% of participants indicated that they routinely receive explicit instructions. Furthermore, 40% of the work authorizations were consistently endorsed by the prescribing dentist. The study's findings mirror those of earlier research [14-15], suggesting that laboratory technicians continue to make significant decisions on the dentist's behalf, a trend that has remained largely unchanged over time.

The prevalent use of acrylic resin and the lower usage of flexible thermoplastic resin polymers suggest areas for further research and potential development. Understanding why these materials are chosen and how they perform could lead to advancements in materials science and denture fabrication techniques, improving the quality of the final product for patients. Only 36% of the laboratories used digital technology to design and fabricate removable partial dentures more than 75% of the time, revealing its limited contribution to the removable prosthesis market. Digital advancements have significantly expedited the design and manufacturing processes of dental products and prostheses. However, these technologies still require human judgment and collaborative effort between technicians and healthcare providers to configure the software parameters, as complete reliance on artificial intelligence for dental prosthetic procedures is not yet viable [16-17].

The survey found that a significant proportion (58%) of technicians encountered challenges regarding work quality and communication during RPD fabrication. This suggests a potential deficiency in the standard of work authorizations and understanding of laboratory procedures imparted to newly graduated dental students, leading to an increased dependence on dental laboratory technicians for expertise.

The survey's results hint at a trend where dentists are ordering laboratory services with insufficient records, thereby placing the dental laboratory in a precarious position. Only about 35% of dentists consistently provided accurate impressions or master casts with adequate extensions capturing vestibules, retromolar pads, the palate, etc. Moreover, only 43% sent high-quality opposing casts or impressions suitable for RPD fabrication. While dental technicians may not always know what properly prepared tooth modifications for partial dentures (such as rest seats and guide planes) should look like, the majority reported that master casts often lacked adequate rest seats or guide planes.

The survey also revealed that the use of the altered or corrected cast impression technique, crucial for supporting distal extension bases, is rarely reported as it necessitates an additional appointment. Typically, dentists opt for the simplest technique or method available when treating patients who require removable prosthodontic restorations. Over 20% of frameworks were returned by the dentist as they did not fit well in the mouth, as reported by 27% of technicians. This reflects discrepancies in the quality of casts and impressions provided by dentists and the quality of restorations made by them. This demands improvements in the concept of dentist-technician communication and integration to further improve the quality of patient care.

Enhancing the engagement of dental technicians in case design and fostering more comprehensive and precise communication from dentists could facilitate a deeper comprehension of case necessities, culminating in superior denture fabrication. This could call for modifications in the educational approach for both dentists and dental technicians, underscoring the significance of collaboration and effective communication. Moreover, the challenges associated with the precision of impressions and the appropriateness of tooth modifications highlight the need for improved quality control procedures, supplementary training, or possibly the invention of novel technologies to enhance these facets of denture production. It's acknowledged that, due to inadequate training, dentists often rely on dental technicians for the design of dental prostheses, a practice that is generally acceptable. However, the design of any prosthesis, which incorporates both mechanical and biological principles, remains the dentist's responsibility. This reliance on dental technicians for prosthesis design, coupled with poor patient compliance, can stem from insufficient dental education [18].

This study also had several limitations that should be acknowledged in the interpretation of its findings. The study relied on self-reported data from dental technicians, which may be subject to recall bias and could potentially overestimate or underestimate the actual perceptions and practices. Objective measures or third-party reports could complement self-reported information in future research. Also, the sample was confined to Saudi Arabia, limiting the generalizability of the findings to other contexts. Different countries and regions may have distinct practices and challenges due to variations in education, regulation, and available resources. Hence, replicating this study in different geographical contexts would be valuable for a broader understanding of the global landscape.

## Conclusions

The findings revealed a significant reliance on acrylic resin, particularly heat-cured, for the fabrication of removable partial dentures, while the usage of flexible thermoplastic resin polymers was less common. It also highlighted some challenges in the communication between dental technicians and dentists, especially while designing the prosthesis to meet the individual needs of the patients concerning case design. The self-report from dental technicians shows gaps in communication with the dentists, suggesting improvements in the quality of impressions and tooth alterations presented to them to achieve a favorable outcome for the prosthesis. The frequency of dentists approaching the technicians to discuss the design of the case varied, with a significant percentage indicating that this only happened occasionally or upon request. While the study was subject to certain limitations, such as its cross-sectional design, reliance on self-reported data, and focus on the Saudi Arabian context, it nonetheless provided valuable insights that can guide future research and practice in the realm of removable partial denture fabrication.
